# Supplementary material for: NR4A1 Ligands as Potent Inhibitors of Breast Cancer Cell and Tumor Growth
Source: Cancers (Basel). 2021 May 29;13(11):2682. doi: 10.3390/cancers13112682 (PMC8198788; doi:10.3390/cancers13112682)
Supplement: Supplementary file 1 [file cancers-13-02682-s001.zip › cancers-1241589-supplementary.pdf]

Supplemental Figure 1

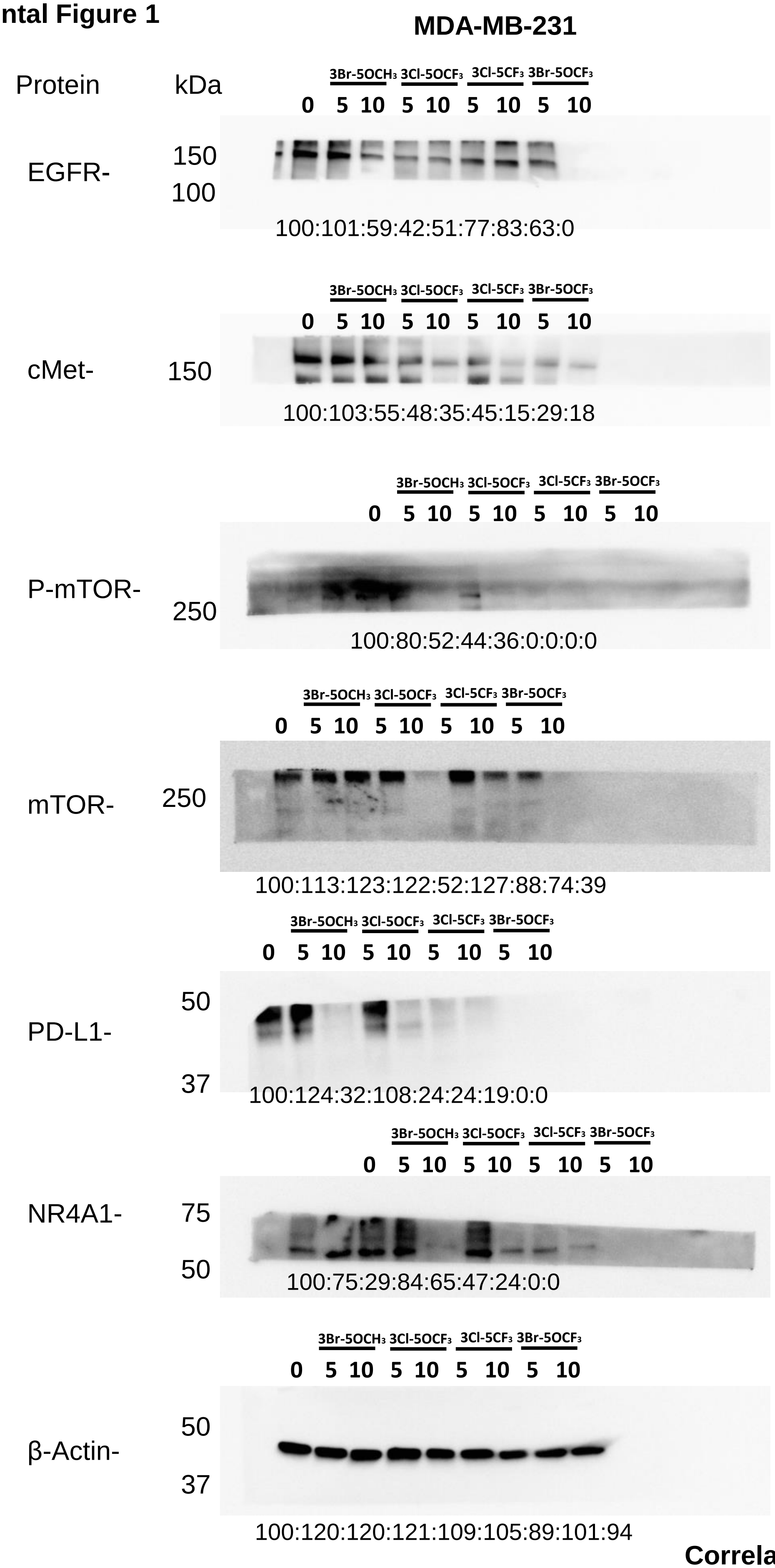

Correlates to Figure 3

Supplemental Figure 2

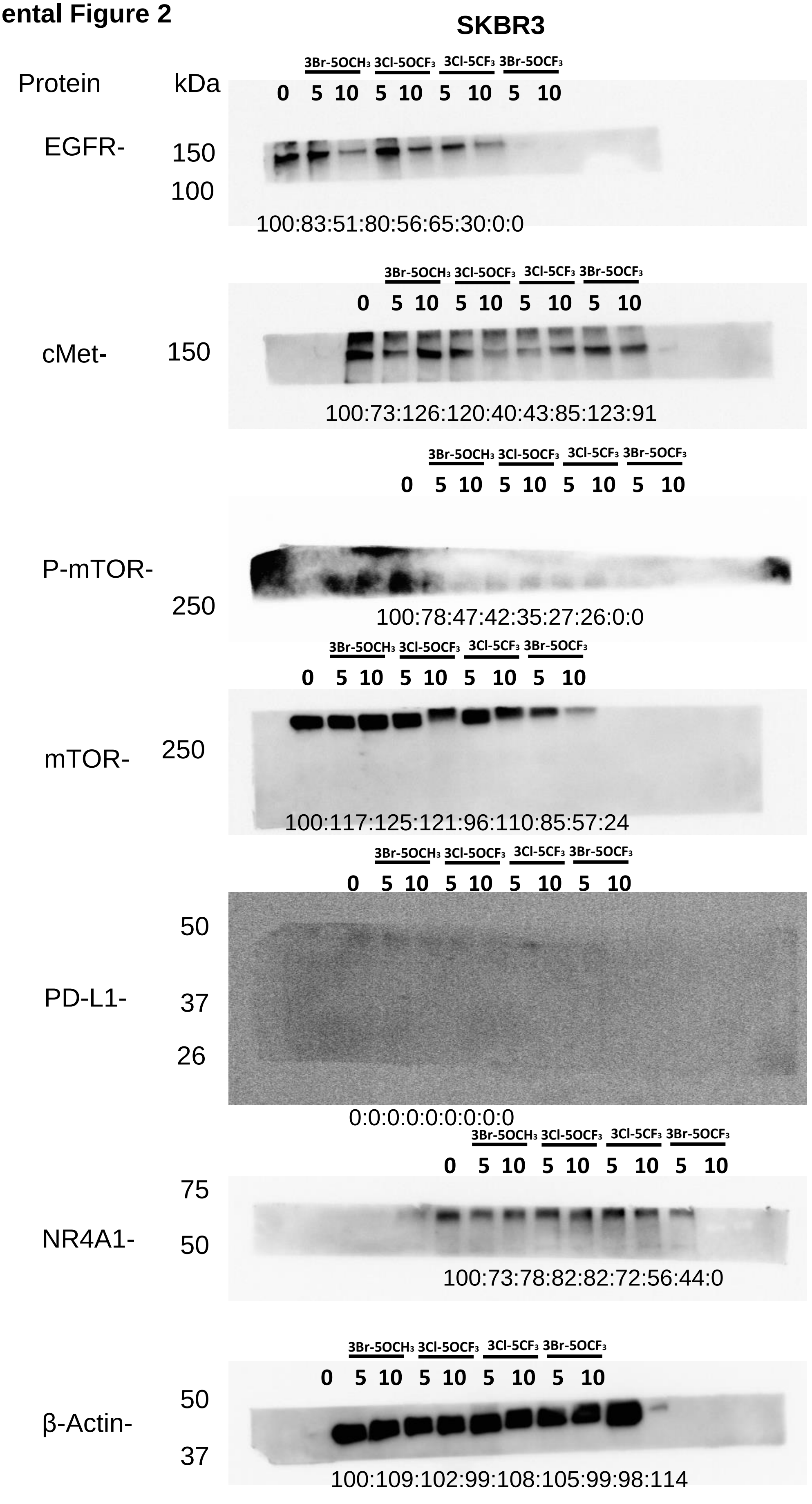

Correlates to Figure 4

Supplemental Figure 3

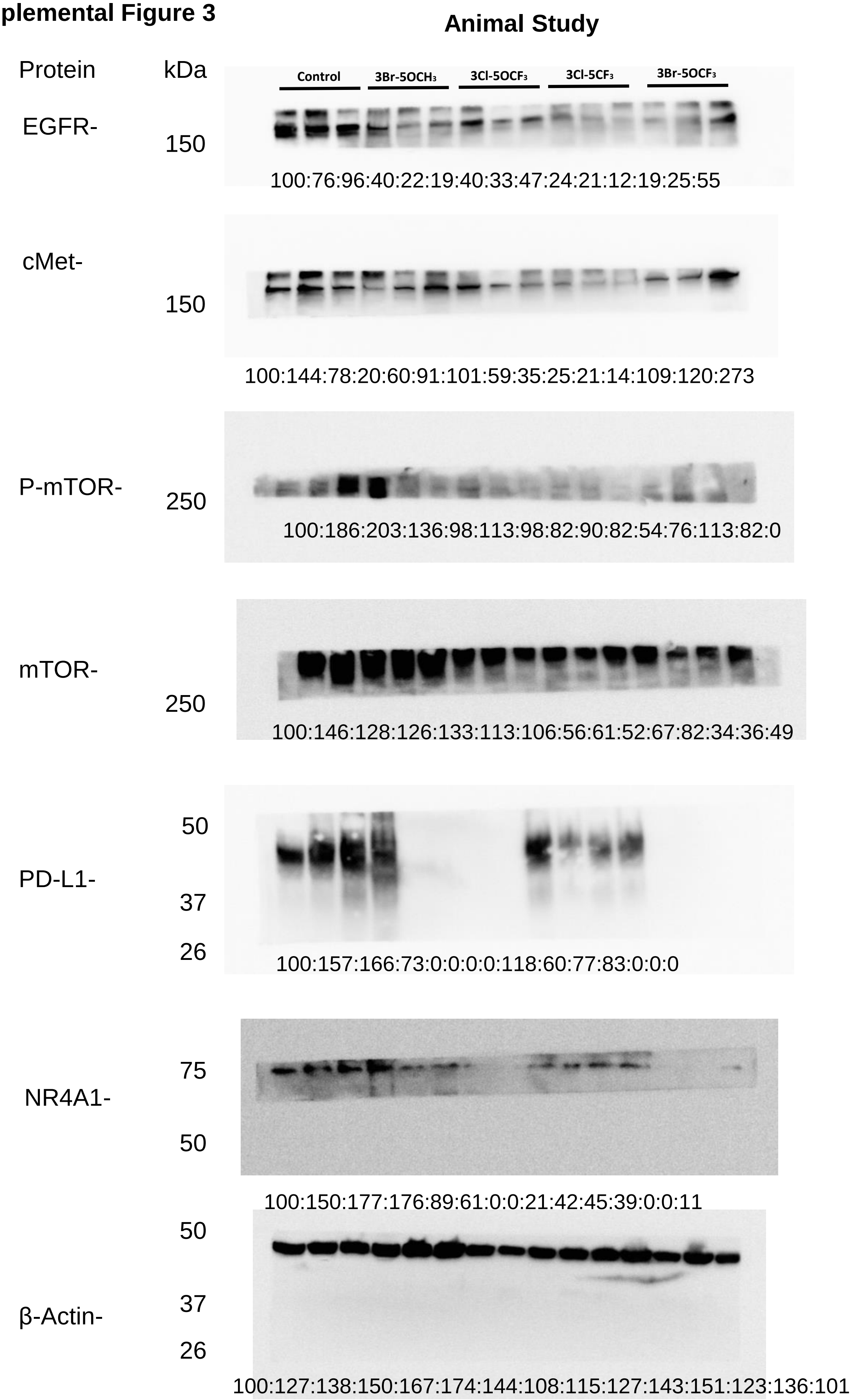

Correlates to Figure 7

Supplemental Figure 4

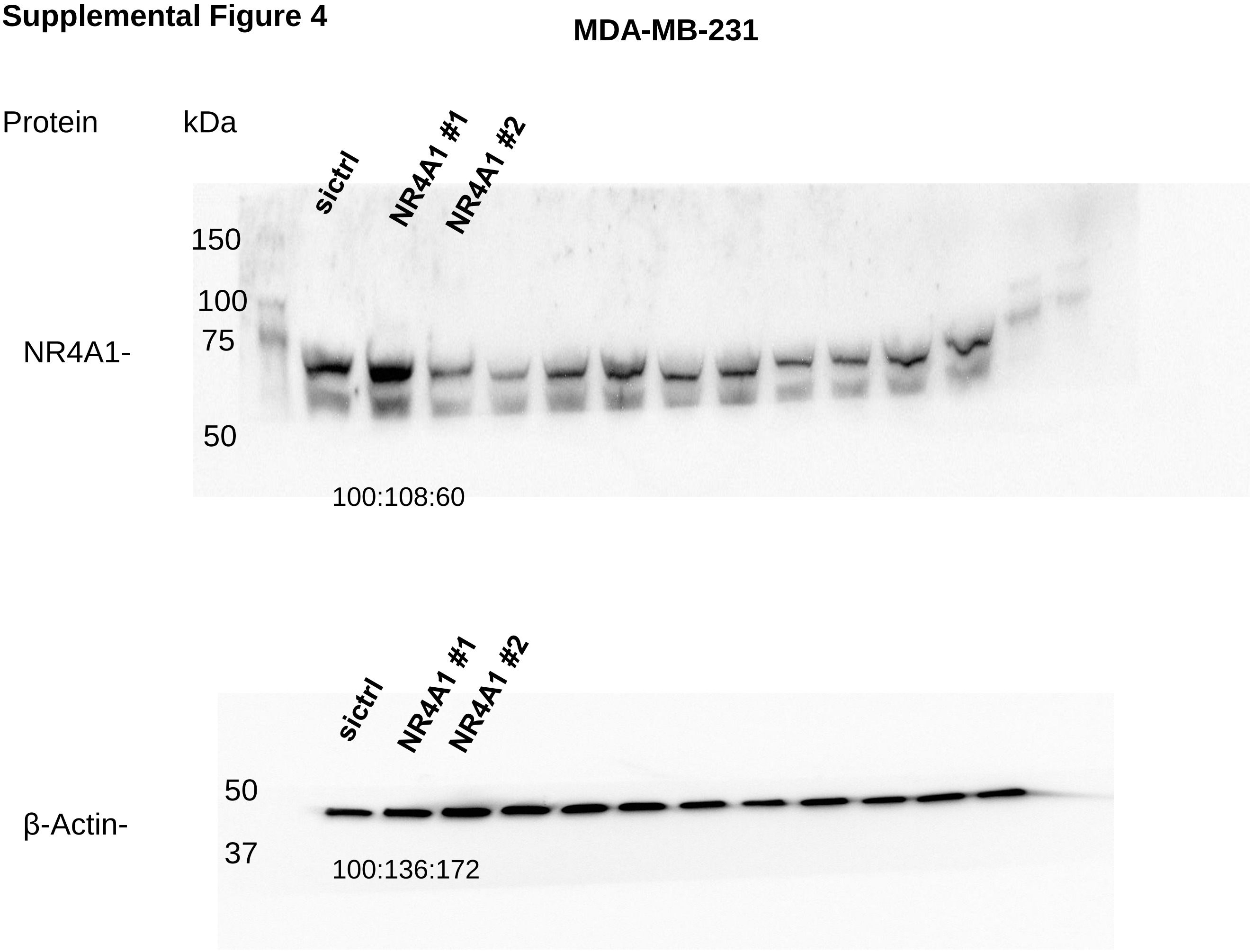

Correlates to Figure 3E

Supplemental Figure 5

SKBR3

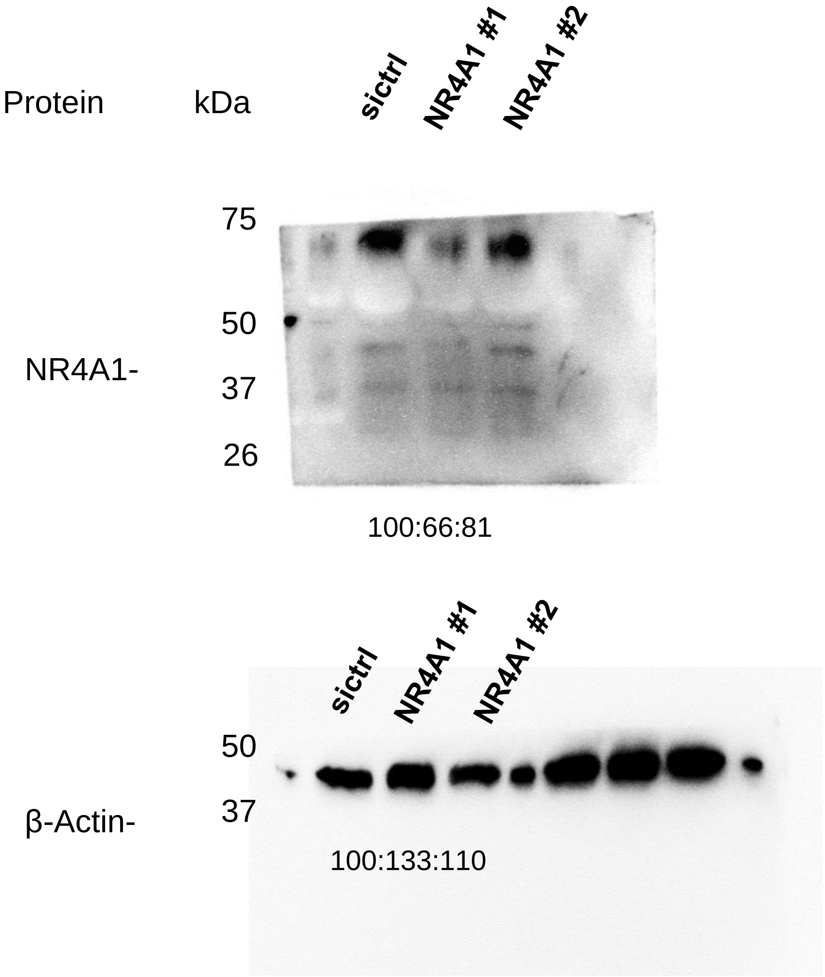

Correlates to Figure 4E
